# PRMT5 Is Upregulated in Malignant and Metastatic Melanoma and Regulates Expression of MITF and p27^Kip1^

**DOI:** 10.1371/journal.pone.0074710

**Published:** 2013-09-30

**Authors:** Courtney Nicholas, Jennifer Yang, Sara B. Peters, Matthew A. Bill, Robert A. Baiocchi, Fengting Yan, Saïd Sïf, Sookil Tae, Eugenio Gaudio, Xin Wu, Michael R. Grever, Gregory S. Young, Gregory B. Lesinski

**Affiliations:** 1 Department of Internal Medicine, Division of Medical Oncology, The Ohio State University Comprehensive Cancer Center, Columbus, Ohio, United States of America; 2 Department of Pathology, Division of Dermatopathology, The Ohio State University Comprehensive Cancer Center, Columbus, Ohio, United States of America; 3 Department of Internal Medicine, Division of Hematology, The Ohio State University Comprehensive Cancer Center, Columbus, Ohio, United States of America; 4 Department of Molecular and Cellular Biochemistry, The Ohio State University, Columbus, Ohio, United States of America; 5 Department of Molecular Virology, Immunology and Medical Genetics, The Ohio State University, Columbus, Ohio, United States of America; 6 The Ohio State University Comprehensive Cancer Center, Columbus, Ohio, United States of America; 7 Center for Biostatistics, Arthur G. James Cancer Hospital and Richard J. Solove Research Institute, The Ohio State University, Columbus, Ohio, United States of America; Duke University Medical Center, United States of America

## Abstract

Protein arginine methyltransferase-5 (PRMT5) is a Type II arginine methyltransferase that regulates various cellular functions. We hypothesized that PRMT5 plays a role in regulating the growth of human melanoma cells. Immunohistochemical analysis indicated significant upregulation of PRMT5 in human melanocytic nevi, malignant melanomas and metastatic melanomas as compared to normal epidermis. Furthermore, nuclear PRMT5 was significantly decreased in metastatic melanomas as compared to primary cutaneous melanomas. In human metastatic melanoma cell lines, PRMT5 was predominantly cytoplasmic, and associated with its enzymatic cofactor Mep50, but not STAT3 or cyclin D1. However, histologic examination of tumor xenografts from athymic mice revealed heterogeneous nuclear and cytoplasmic PRMT5 expression. Depletion of PRMT5 via siRNA inhibited proliferation in a subset of melanoma cell lines, while it accelerated growth of others. Loss of PRMT5 also led to reduced expression of MITF (microphthalmia-associated transcription factor), a melanocyte-lineage specific oncogene, and increased expression of the cell cycle regulator p27^Kip1^. These results are the first to report elevated PRMT5 expression in human melanoma specimens and indicate this protein may regulate MITF and p27^Kip1^ expression in human melanoma cells.

## Introduction

The incidence of melanoma is rising faster than that of any other cancer [1]. Although early stage disease can be treated with surgery, advanced melanoma remains devastating, with five-year survival approximately 15% [2]. Recent progress in targeted and Immunomodulatory therapies such as vemurafenib (Zelboraf, BRAF^V600E^ inhibitor) and ipilimumab (Yervoy, anti-CTLA4 therapy) have improved outcomes for patients with metastatic melanoma. Despite these advances, few alternative therapies exist for advanced melanoma, and these available treatments work for only a limited time. Thus there is a need for a greater understanding of melanoma biology and new treatments.

Post-translational protein modification is involved at all levels of cellular regulation. Protein arginine methyltransferases (PRMT) catalyze the attachment of methyl (CH_3_) groups to the guanidino nitrogen atoms of arginine amino acid residues. Currently, eleven PRMT proteins have been identified in humans. These proteins are homologous and share a central catalytic domain. PRMT enzymes methylate their targets in either an asymmetric (Type I PRMTs) or symmetric configuration (Type II) [3]. Increased protein levels of PRMT5 have been observed in leukemia, lymphoma, glioma, ovarian, breast, prostate, and lung cancer. Therefore, these enzymes are generating increased interest as therapeutic targets [4]–[12]. To date, their expression in human melanoma specimens has not been explored, and their biologic role in melanoma is not fully understood.

PRMT5, a Type II arginine methyltransferase regulates cellular functions including apoptosis, Golgi structure, pluripotency, cell growth, and snRNP biosynthesis [4], [6], [7], [13]–[26]. Although prior studies provide insight into mechanistic features of PRMT5, most data are derived from a limited panel of cell lines. PRMT5 likely plays a unique role across individual tumor types. Indeed, PRMT5 over-expression can influence progression of leukemia, lymphoma, glioma, breast, prostate and lung cancer [4]–[11]. Reports also demonstrate that PRMT5 regulates ERK signal transduction amplitude in *BRAF* wild type melanoma cell lines [18]. While there are several PRMT family members, PRMT5 is distinct among them because of its apparent role in promoting a malignant phenotype. To date there is only one known form of the PRMT5 protein, though two mRNA isoforms exist which differ in the second intronic region. This is in contrast with several other PRMT family members which can have as many as four protein forms. Finally, though PRMT10 and PRMT11 show sequence homology to the other PRMTs, they have not yet demonstrated methyltransferase activity *in vitro* or *in vivo* [3]. For these reasons, we postulated that PRMT5 deserved further investigation in melanoma.

PRMT5 is classically associated with chromatin remodeling, gene expression, and has an essential role in embryogenesis [26]. Based on these properties, it was of interest for us to determine whether any relationship was evident between PRMT5 expression and microphthalmia-associated transcription factor (MITF). Indeed, MITF represents a transcription factor which regulates melanomagenesis and tumor formation, and is an essential regulator of melanocyte proliferation and differentiation. Furthermore, increased *MITF* copy number has been documented in metastatic tumors, and correlates with decreased 5-year survival in metastatic melanoma [27]. MITF has been implicated in melanocyte cell survival mechanisms, including regulation of several cyclin-dependent kinase inhibitors (CKI) p16^INK4a^, p21^Waf1/Cip1^, and p27^Kip1^ [28]–[30]. However, no publications to date have explored PRMT5 or its relationship with MITF and cell cycle regulatory proteins in primary melanoma specimens.

We hypothesized that PRMT5 plays a role in regulating growth of human melanoma. Here, we demonstrate that PRMT5 expression was significantly elevated in melanocytic cells with dysregulated growth. In patient specimens, PRMT5 was upregulated in melanocytic nevi, malignant, and metastatic melanoma, compared to normal epidermis. In human metastatic melanoma cell lines, PRMT5 was predominantly cytoplasmic, and associated with its cofactor Mep50. However, histologic examination of tumor xenografts revealed a heterogeneous pattern of nuclear and cytoplasmic PRMT5 expression. PRMT5 regulated melanoma cell growth, as siRNA-mediated PRMT5 depletion inhibited proliferation in a subset of metastatic melanoma cell lines (including Hs294T, 1106Mel, WM1366, and CHL-1) but accelerated growth of others (A375, MeWo). PRMT5 depletion also reduced MITF expression, and increased p27^Kip1^. These results represent the first characterization of PRMT5 expression in human melanoma specimens and indicate this protein may regulate growth and the expression of MITF and p27^Kip1^ in human melanoma cell lines.

## Results

### PRMT5 protein is upregulated in human melanoma

**Figure 1:**
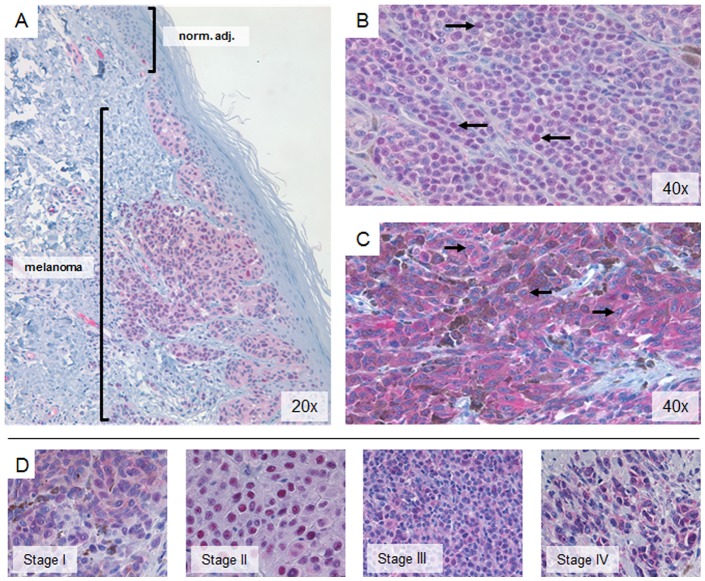
PRMT5 is upregulated in human malignant melanoma tumors compared to normal epidermis, and subcellular localization of PRMT5 is heterogeneous amongst patient tumors. A, Brightfield image of PRMT5 staining in a Stage 2/3 tumor and its adjacent normal epidermis. PRMT5 protein was visualized with Vulcan FastRed label (pink); samples were counterstained with hematoxylin to delineate nuclei (arrows) and connective tissue (blue). B, malignant skin tumor of pathologic stage 4, showing predominantly nuclear PRMT5. C, malignant skin tumor of pathologic stage 4, showing predominantly cytoplasmic PRMT5 (arrows indicate hematoxylin-stained nuclei). D, representative images of PRMT5 staining in patient tumors of pathologic stages I–IV.

PRMT5 protein expression in human melanoma specimens was evaluated by IHC in 248 de-identified tissues from individual patient samples and two separate tissue microarrays. Clinical outcomes data was not available for these samples. Included in these samples were normal skin, melanocytic nevi, primary malignant melanoma tumors (*in situ* and Clark's stage I-IV), and metastatic melanoma tumors (Table S1). Initially, the overall percentage of PRMT5 positive (PRMT5+) cells in each specimen was assessed. Samples containing ≤5% PRMT5+ cells were scored negative, while samples with ≥5% PRMT5+ cells were positive. Overall, the number of samples with ≥5% PRMT5+ cells was significantly higher in malignant (121 of 135 samples PRMT5+) and metastatic (58 of 66 samples PRMT5+) melanoma specimens versus normal epidermis (1 of 21 samples PRMT5+; p<0.0001) (Table 1 and Figure 1A). Many clinically relevant molecular changes in melanoma (e.g., *BRAF* ^V600E^ mutation, STAT3 Tyr^705^ phosphorylation) have also been detected in melanocytic nevi, suggesting early events trigger aberrant growth or transformation [31]–[33]. Therefore, we analyzed a small heterogeneous group of proliferative melanocytic nevi, and observed significantly increased PRMT5+ cells versus normal skin (23 of 26 melanocytic nevi and 1 of 21 normal epidermis samples were PRMT5+, p<0.0001).

**Table 1:** The arginine methyltransferase PRMT5 is significantly overexpressed in human melanocytic nevi, malignant, and metastatic tissues compared to normal epidermis.

	number (and %) of patient specimens negative or positive for PRMT5		
% of PRMT5 positive cells per sample:	<5% (neg)	≥5% (pos)	Total
*normal epidermis	20 (95%)	1 (5%)	21
melanocytic nevi	3 (12%)	23 (88%)	26
malignant	14 (10%)	121 (90%)	135
metastatic	8 (12%)	58 (88%)	66

* *p<0.001 compared to melanocytic nevi, malignant, and metastatic tissue, Fisher's exact test.*

The staining intensity of PRMT5 was also assessed. Due to heterogeneity of tissue quality and processing, data from each of two tissue arrays were analyzed independently. Overall, there was no difference in staining intensity between tissue types (normal epidermis, melanocytic nevi, malignant, or metastatic melanomas). However, a positive correlation between staining intensity and the percentage PRMT5+ cells was observed in the two arrays. This was true for both nuclear (Spearman correlation 0.79 (array 1) and 0.80 (array 2), *p*<0.0001) and cytoplasmic staining (Spearman correlation 0.59 (array 1) and 0.81 (array 2); *p*<0.0001) (not shown).

### Localization of PRMT5 in patient specimens and cell lines

Marked heterogeneity in PRMT5 localization was observed across patient samples (Figure 1B–D). Regardless of stage, some patient specimens displayed predominantly nuclear (Figure 1B), or cytoplasmic staining (Figure 1C). Despite this heterogeneity, the percentage of nuclear PRMT5+ cells was significantly lower in metastatic tumors compared to primary cutaneous tumors (Table 2; p<0.0001). Additionally, melanoma tissue displayed significantly higher cytoplasmic PRMT5+ cells as compared to normal epidermis (p<0.0001), but a lower percentage of cytoplasmic PRMT5+ cells when compared to melanocytic nevi (Table 3; p<0.0144).

**Table 2:** The distribution of nuclear PRMT5 protein differs significantly among normal epidermis, benign nevi, and malignant and metastatic tumors.

	number (and %) of patient specimens in each category				
% of cells per specimen which are nuclear positive:	0–5% (neg)	6–25%	26–75%	76–100%	Total
normal epidermis^¶†$^	20 (95%)	0 (0%)	1 (5%)	0 (0%)	21
melanocytic nevi ^♦¶$^	1 (4%)	4 (7%)	7 (29%)	12 (50%)	24
malignant ^♦†$^	24 (18%)	32 (24%)	59 (44%)	18 (14%)	133
metastatic ^♦†¶^	23 (35%)	18 (29%)	22 (33%)	2 (3%)	66

*p<0.001 in comparison with normal epidermis (♦), melanocytic nevi (†), malignant (¶), metastatic ($), Wilcoxon rank sum test.*

**Table 3:** The distribution of cytoplasmic PRMT5 protein differs significantly among normal epidermis, benign nevi, and malignant and metastatic tumors.

	number (and %) of patient specimens in each category				
% of cells per specimen which are cytoplasmic positive:	0–5% (neg)	6–25%	26–75%	76–100%	Total
normal epidermis^¶†$^	19 (95%)	0 (0%)	1 (5%)	0 (0%)	20
melanocytic nevi ^♦¶^	6 (20%)	0 (0%)	4 (16%)	16 (64%)	25
malignant ^♦†^	34 (26%)	14 (11%)	45 (35%)	36 (28%)	129
metastatic ^♦^	19 (32%)	2 (3%)	17 (29%)	21 (36%)	59

*p<0.05 in comparison with normal epidermis (♦), melanocytic nevi (†), malignant (¶), metastatic ($), Wilcoxon rank sum test.*

**Figure 2:**
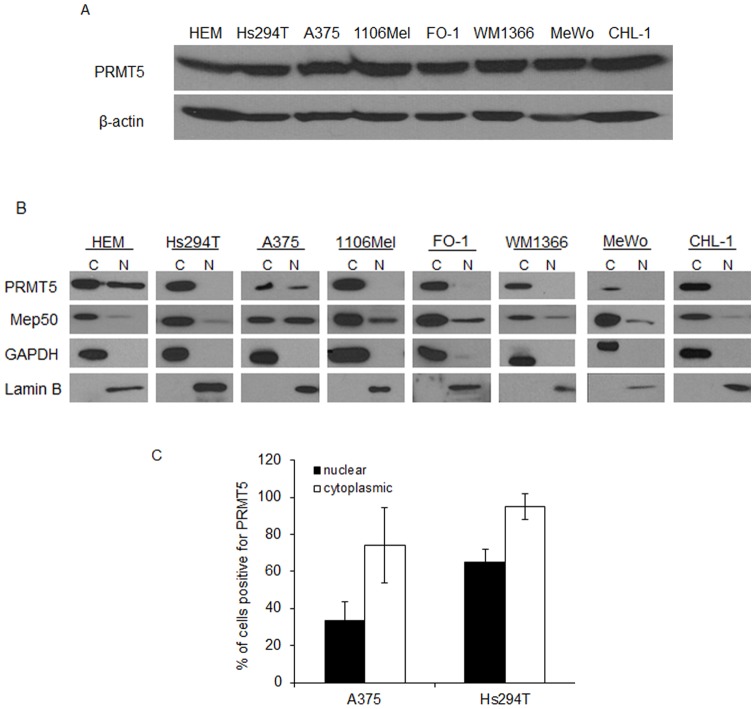
PRMT5 is present and displays heterogeneous localization between human cell lines and orthotopic tumors. A, Detection of PRMT5 in cell lysates from immortalized human epidermal melanocytes (HEM) or a panel of human metastatic melanoma cell lines. β-actin was used as loading control. B, HEM and melanoma cell lines underwent subcellular fractionation and immunoblot analysis for PRMT5, Mep50, or markers for nuclear (lamin B) and cytoplasmic (GAPDH) fractions. C, 1×10^6^ cells from the A375 and Hs294T human melanoma cell lines were implanted into the flank of immunocompromised mice and grown for 21 days. Tumors were removed, fixed in formalin, and analyzed via IHC for PRMT5. Data represent total percentage of cells in each tumor positive for PRMT5 in nucleus, cytoplasm, or both. n≥2 tumors were analyzed.

A series of studies was conducted in a panel of human metastatic melanoma cell lines to explore the role of PRMT5 in melanoma biology. PRMT5 expression was detectable in cell lines derived from diverse organ sites and with unique genetic profiles, as well as immortalized human epidermal melanocytes (HEM) (Figure 2A). All lines expressed PRMT5 irrespective of *BRAF* or *NRAS* mutational status (Table 2). Subcellular fractionation revealed that PRMT5 was present in both the nucleus and cytoplasm of immortalized melanocytes, but was predominantly cytoplasmic in metastatic lines (Figure 2B). Of the lines examined, only A375 cells expressed detectable nuclear PRMT5.

To examine if the tumor microenvironment impacts PRMT5 localization *in vivo*, A375 or Hs294T cells were implanted subcutaneously into athymic mice. IHC analysis confirmed PRMT5 expression in tumors from both cell lines. In both A375 and Hs294T tumors, PRMT5 expression was highest in the cytoplasm (Figure 2C). However, in both cell lines, a large proportion of cells (range  = 35–65%) were also positive for PRMT5 in the nucleus. These data suggest that the tumor microenvironment may impact PRMT5 localization in melanoma cells.

### PRMT5 associates with Mep50, but not with cyclin D1 or STAT3, in melanoma cell lines

**Figure 3:**
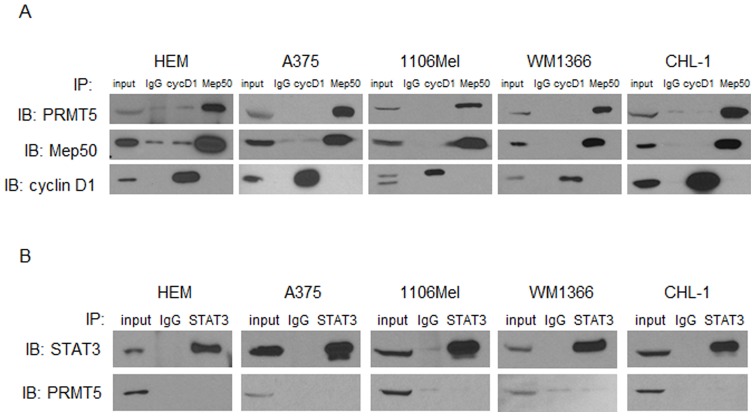
PRMT5 associates with Mep50, but not cyclin D1 or STAT3 in a subset of melanoma cell lines. A, HEM and representative human melanoma cell lines were harvested and subjected to total cell lysis followed by immunoprecipitation. 10% of input protein, as well as protein from IgG control and IP directed against Mep50 and cyclin D1, underwent immunoblot analysis for IP target proteins as well as PRMT5. B, IP against STAT3 was performed on the same cell line panel, and subjected to immunoblot analysis using antibodies against STAT3 or PRMT5.

Recent studies have documented a physical interaction between PRMT5, its enzymatic co-factor Mep50, STAT3, and cyclin D1 [26], [34], [35]. We determined whether these interactions also occurred in melanoma or if they were unique to the model systems in these previously published works. Indeed, Mep50, cyclin D1 and STAT3 were expressed in melanoma lines, and Mep50 was predominantly cytoplasmic (Figure 2B; data not shown). Immunoprecipitation experiments revealed that PRMT5 associated with Mep50, but not cyclin D1 or STAT3 in these cell lines (Figure 3A–B). PRMT5 also did not associate with cyclin D1 in either unsynchronized melanoma cells, or cells harvested in G1 or G2/M phases following double thymidine block (not shown).

### siRNA-mediated PRMT5 depletion modulates proliferation and expression of p27^Kip1^ and MITF

**Figure 4:**
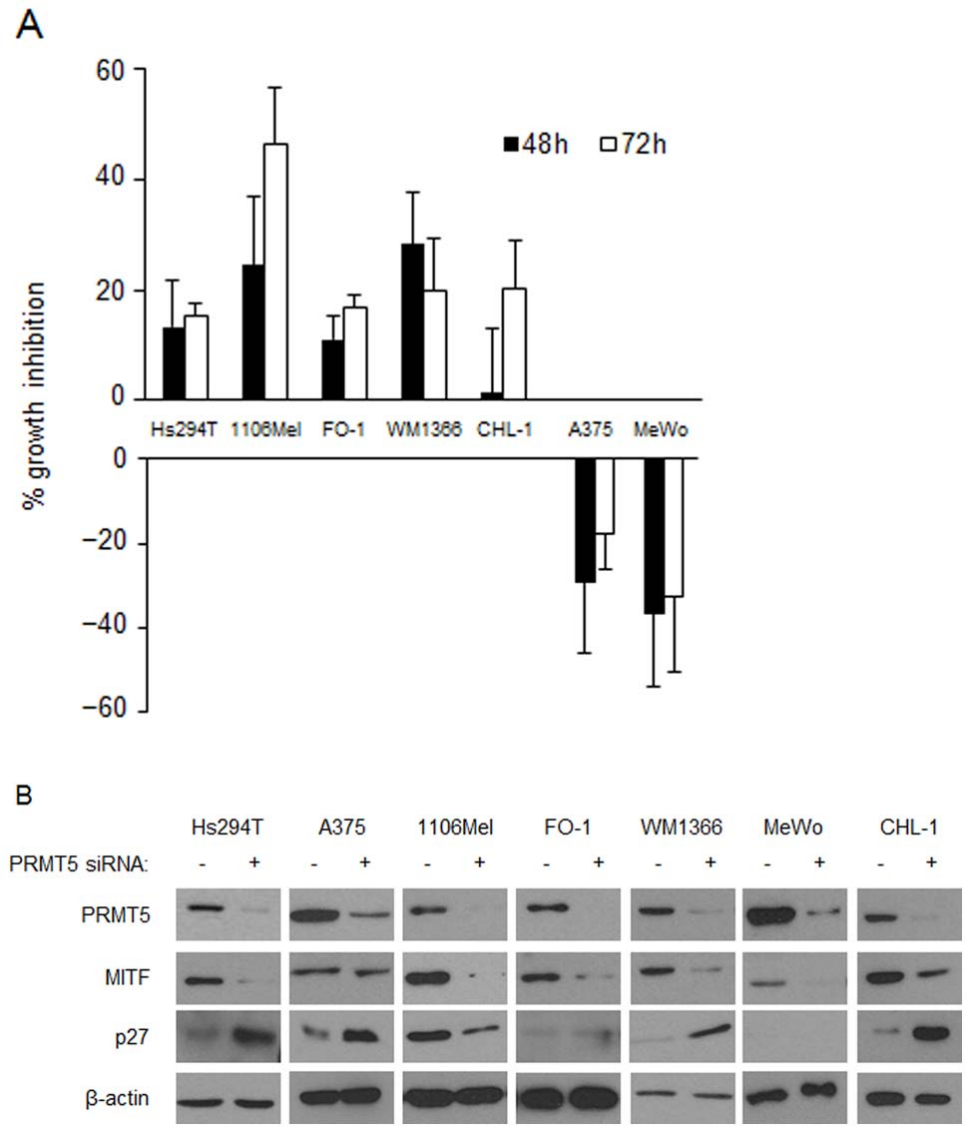
Depletion of PRMT5 via siRNA modulates cell proliferation and expression of MITF and p27^Kip1^. A, Melanoma cell lines were transiently transfected with scrambled siRNA or siRNA targeting PRMT5. Cell growth was measured by MTT assay at 48-transfection. Data represent percent of growth inhibition ± SEM (n≥3). B, Analysis of PRMT5, MITF, p27^KIP1^, and β-actin protein expression at 48 hours post-transfection. n≥2 transfections were performed per cell line; representative immunoblots are shown here. The MITF antibody used here for IB predominantly recognizes one of the two known MITF isoforms in human melanocytes.

To determine how PRMT5 loss affects melanoma function, we utilized PRMT5-targeting siRNA [15]. Melanoma cell lines were transfected with PRMT5 siRNA or scrambled negative control sequence. PRMT5 siRNA decreased protein expression at 24, 48, and 72 h, but did not induce apoptosis as measured by PARP cleavage and lack of non-adherent cells (not shown). PRMT5 depletion in 1106Mel, FO-1, Hs294T, and WM1366 lines inhibited growth at 48 h (11–28% mean inhibition) and 72 h (15–46% mean inhibition), versus scrambled siRNA (Figure 4A). Interestingly, PRMT5 depletion in A375 and MeWo cell lines increased proliferation at 48h (29% and 37%, respectively) and 72 h (18% and 33%, respectively). Due to the heterogeneous genotypic profile in human melanoma, there was no clear relationship between *BRAF or NRAS* that predicted effects of PRMT5 loss on proliferation (Table S2; and Figure 4A). However, PRMT5 depletion reduced expression of MITF protein in 6 of 7 of cell lines (Figure 4B). Consistent with prior reports in MITF-depleted cells [30], p27^Kip^ expression increased in 5 of 6 cell lines, irrespective of *BRAF* or *NRAS* status (Figure 4B). We also determined that MITF is not likely an enzymatic target of PRMT5 as it did not co-precipitate with PRMT5 (Figure S1), nor was it symmetrically dimethylated (data not shown). Finally, PRMT5 loss did not consistently regulate MITF or p27^Kip1^ at the transcriptional level, or alter specific microRNAs that are key regulators of these proteins including miR-221, miR-222, miR-181b or miR-148a [36]–[39] (Table 4 and data not shown). These data indicate that PRMT5 plays a role in modulating the key proteins MITF and p27 which are relevant to melanoma cell biology and proliferation.

**Table 4:** Effects of PRMT5 siRNA on microRNA (miR) expression in human melanoma cell lines.

Fold Change vs. Control siRNA				
Cell Line	miR-221	miR-222	miR-181b	miR-148a
Hs294T	1.62	1.29	1.02	1.21
WM1366	0.84	0.67	1.39	0.63
CHL-1	0.42	0.74	0.68	2.38
1106 MEL	1.16	1.42	2.38	0.72

## Discussion

In this report, we demonstrate for the first time that PRMT5 is upregulated in primary human malignant and metastatic melanoma specimens, as well as in melanocytic nevi. Using human cell lines, we further demonstrate that siRNA-mediated loss of PRMT5 led to reduced MITF expression, increased p27^Kip1^ expression and variable effects on proliferation. These data indicate a unique role for PRMT5 in regulating proteins relevant to melanoma biology and proliferation.

Numerous proteins interact directly with PRMT5 or are methyltransferase targets. These interactions regulate many essential cellular processes including proliferation, apoptosis, gene expression and tumor suppression [5], [15], [18], [23], [26], [34], [40]–[45]. Furthermore, PRMT5 over-expression occurs in glioblastoma, leukemia, lung, ovarian, and prostate cancer and lymphoma [4], [8]–[12], [43], [46], [47]. However, few if any of these interactions have been validated in melanoma. Data from this study indicate that although PRMT5 is expressed at high levels, previously published protein-protein interactions may differ in melanoma when compared to those in other malignancies.

PRMT5 protein was significantly increased in both cytoplasmic and nuclear compartments of malignant and metastatic melanomas, as well as in melanocytic nevi. This suggests that PRMT5 expression may fluctuate during progression, and PRMT5 upregulation may be an early event in melanomagenesis. It is possible that PRMT5 may cooperate with other factors that have been shown to contribute to the formation of premalignant lesions including *BRAF* mutations, *MITF* overexpression, DNA damage and nonfunctioning nucleotide excision repair, and more recently the observation that macrophages and melanin also play a role in tumor initiation [48], [49]. Since *BRAF* mutations, changes in *MITF* expression, and constitutive STAT3 activation are detected in pre-malignant melanocytic nevi [32], [50], PRMT5 expression and activity in early lesions may be prognostic and also deserves further investigation. Due to the limited clinical information available on patient specimens, and retrospective nature of this initial study, we were not able to explore the impact of ultraviolet light exposure, or prior targeted or chemotherapies as potential modifiers of PRMT5 expression in patient samples. This is relevant given the recently described role for PRMT5 in controlling keratinocyte differentiation through the MAPK pathway [51].

The pattern of subcellular localization of this protein was strikingly different among many patient samples. Interestingly, the percentage of nuclear PRMT5+ cells was lower in metastatic compared to malignant tissue, but higher in melanocytic nevi compared to malignant tissue (Table 2). These data suggest that PRMT5 localization may be a dynamic process that occurs during melanoma progression. Our observations that PRMT5 was predominantly localized in the cytoplasm of cell lines were consistent with data showing higher cytoplasmic expression in metastatic tumors (Table 3). Although PRMT5 localization in patient specimens was more heterogeneous, it is important to note that patient tumors represent tissue exposed to various factors within the microenvironment including a milieu of cytokines, integrin interactions and hypoxic regions [52], [53]. Similarly, orthotopic tumors in mice (Figure 2C) also showed a heterogeneous localization of PRMT5 in comparison to the parent cell line growing *in vitro*. *In vitro* studies do not account for interactions with surrounding tissues or immune cells. The influence of these factors on PRMT5 localization will be an important area of future investigation.

The cytoplasmic predominance of PRMT5 suggests that its role is not exclusive to control of gene expression in melanoma. This observation was consistent with cytoplasmic PRMT5 expression in a subset of other tumor types including lung and prostate cancer [10], [11]. Immunoprecipitation experiments further revealed that PRMT5 co-precipitated with Mep50, but not with cyclin D1 or STAT3. Ultimately the lack of interaction between PRMT5 and cyclin D1 or STAT3, and differences in subcellular localization, suggest that the role of PRMT5 in melanoma may differ from its role in other cancers.

We next tested the effects of PRMT5 loss on proliferation and relevant pathways in melanoma cell lines. Depletion of PRMT5 resulted in growth rate inhibition in Hs294T, 1106Mel, FO-1, WM1366, and CHL-1, but acceleration of the growth rate in A375 and MeWo cells. Though technical challenges prevented the complete depletion of PRMT5 protein in several cell lines including A375 and MeWo, there was trace PRMT5 detected in several other cell lines, and therefore is not likely the explanation for differences in proliferation. More likely it is the influence of heterogeneous genotypes between these cell lines that contribute at least partially to the divergent changes in proliferation (Table S2).

As depletion of PRMT5 resulted in profound effects on cell proliferation, we initially focused on the MITF transcription factor. Previous studies have characterized MITF as a master regulator of melanocyte proliferation, differentiation, and melanoma tumor and stem cell biology [54], [55]. In this study, PRMT5 depletion was associated with decreased MITF in a majority of human melanoma cell lines. However, the mechanism for this observation is likely complex. It is known that MITF expression is regulated by complicated, redundant transcriptional mechanisms and by post-translational modifications regulating protein stability [56]. Consistent with prior studies showing that depletion of MITF leads to increased p27^Kip1^ protein [30], the expression of p27^Kip1^, was increased when MITF was downregulated in cells depleted of PRMT5 via siRNA. In light of these observations, it is likely that PRMT5 loss may affect both MITF and p27^Kip1^ expression through several indirect mechanisms including other pathways that regulate MITF stability via phosphorylation and/or ubiquitination [30], [56].

To date, only one report has published a role for PRMT5 in melanoma, in which PRMT5 was shown to modulate the ERK pathway in *BRAF* wild type cells. PRMT5 was also shown to associate with CRAF protein in PC12 and Cos-7 cells [18]. Our data demonstrated that PRMT5 did not associate with CRAF in unstimulated wild-type or *BRAF^V600E^* mutant melanoma cell lines (Figure S2). Further, we observed that CRAF was not symmetrically dimethylated in unstimulated melanoma cells regardless of genotype (data not shown). Several differences exist between these experiments and the previous report. Namely, in the prior report [18], PRMT5 was shown to associate with CRAF in PC12 and Cos7 cells, and modulate ERK signaling in *BRAF* wild type melanoma cells activated with human growth hormone. Together, these studies indicate that PRMT5 may play diverse roles in regulating melanoma cell biology and is prone to influence by exogenous stimuli.

In the future our studies will focus on examining the relationship between PRMT5 expression and tumor stage, or between primary tumors and biopsies from locally positive sentinel lymph nodes, in order to identify if PRMT5 could be a prognostic marker. In light of the fact that *BRAF* status in melanoma is a predictor of response to BRAF inhibitors, it would also be important to examine the expression and localization of PRMT5 in BRAF inhibitor-resistant versus inhibitor-sensitive cells, or in tumors which are or are not responsive to immunotherapy. These studies will help to clarify the predictive value of PRMT5 in melanoma. In addition, prior studies have suggested, NRAS and CRAF contribute to cell survival in *BRAF* wild-type cells [57]. Thus, it will be important to examine the relationship between PRMT5 and CRAF in *NRAS* wild type and mutant cells which have been exposed to BRAF inhibitors.

In conclusion, we demonstrate that PRMT5 is expressed in human melanoma and melanocytic nevi. Although it was expressed predominantly in the cytoplasm of human melanoma cell lines, clinical and orthotopic tumor data indicate that cellular localization of PRMT5 may be modulated by the tumor microenvironment. PRMT5 also appears to play a unique role in regulation of cell growth and key factors including MITF and p27^Kip1^. Thus, the interactions of PRMT5 with other proteins are likely to be unique in human melanoma cells. Together these results highlight important factors that deserve consideration, as therapeutic strategies move toward targeting PRMT5 or other methyltransferases in melanoma.

## Materials and Methods

### Cell lines and Reagents

Human melanoma cell lines were obtained from the ATCC (A375, Hs294T, MeWo, CHL-1; Manassas, VA), Dr. Soldano Ferrone (1106Mel, FO-1; University of Pittsburgh) or Dr. Meenhard Herlyn (WM1366; Wistar Institute). Immortalized human epidermal melanocytes (HEM) were purchased from ScienCell Inc. (San Diego, CA). The following antibodies were used for immunohistochemistry (IHC), immunoblot (IB), or immunoprecipitation (IP): PRMT5 (Abcam # ab31751; Cambridge, MA), PRMT5 (Cell Signaling Technology #2252; Danvers, MA), β-actin (Sigma # A5441-2; St. Louis, MO); GAPDH (Sigma # G8795); lamin B (Santa Cruz # sc-6216; Santa Cruz, CA), MITF (Thermo-Scientific # MS-772-PO; Lafayette, CO), MITF (Spring Bioscience # E17904; Pleasanton, CA), cyclin D1 (Abcam # ab6152), cyclin D1 (Cell Signaling Technology #2978), Mep50 (Bethyl Laboratories # A301561A; Montgomery, TX), Mep50 (Abnova #79804; Taipei, Taiwan), STAT3 (Cell Signaling Technology #9132), and p27^Kip1^ (Cell Signaling Technology #2552).

### Melanoma patient specimens

All studies were approved by the Institutional Review Board of The Ohio State University Wexner Medical Center (#20100071; Lesinski; #2002H0089; Peters). PRMT5 IHC data were obtained from 21 normal epidermis (including melanocytes and keratinocytes), 26 melanocytic nevi, 135 malignant, and 66 metastatic melanoma human specimens. Formalin-fixed paraffin embedded samples were obtained as commercially available tissue microarrays (Tissue Array Network Inc, http://www.tissue-array.net), arrays from the AIDS and Cancer Specimen Resource (University of California, San Francisco AIDS and Cancer Specimen Resource (ACSR) (http://acsr.ucsf.edu)), or de-identified specimens from OSUWMC. Regarding specimens from the Ohio State University Wexner Medical Center, the need for patient consent was waived by the Institutional Review Board, as no patient identifiers were associated with the tissues. All animal studies were approved by The Ohio State University Institutional Animal Care and Use Committee (IACUC).

### Immunohistochemical analysis of PRMT5 expression

Slides were deparaffinized and rehydrated through graded alcohols and xylene. Antigen retrieval was performed in a solution of citrate buffer in a vegetable steamer. PRMT5 (Abcam) was used at a dilution of 1∶70. The secondary detection system used was Mach 4 Alkaline Phosphatase (Biocare Medical; Concord, CA) with Vulcan Fast Red chromogen (Biocare Medical). Serial sections were stained in duplicate on a Dako Autostainer (Dako; Carpinteria, CA), counterstained (hematoxylin), and mounted. Each specimen was analyzed in a blinded fashion under direction of a board-certified dermatopathologist (S. Peters, M.D.). The percentage of PRMT5+ cells in each sample was recorded, then sorted into four categories: negative (0–5% of cells in sample), low (6–25%), medium (26–75%), or high (>75%) percentage of PRMT5+ cells. Samples were sub-categorized via percentage of PRMT5+ in nucleus or cytoplasm. PRMT5 staining intensity in the nucleus and cytoplasm was qualitatively assessed. PRMT5 negative samples were recorded as zero; positive samples were scored from 1 to 5, where 1 =  low intensity and 5 =  highest intensity. Serial sections from arrays and specimens were stained in duplicate. Twenty-two specimens were discarded due to inadequate, lost, or damaged tissue, poor quality, or discordant results between duplicates as determined by the dermatopathologist.

### Immunoblot analysis

Cell lysates were subjected to immunoblot analysis as described [58], and probed with antibodies (Ab) to human PRMT5, MITF, Mep50, Cyclin D1, STAT3, GAPDH, lamin B, or β-actin. Following incubation with horseradish-peroxidase-conjugated secondary Ab, blots were developed using the West Pico Substrate (Thermo Fisher Scientific).

### Subcellular fractionation

Subcellular fractionation was performed using the NE-PER buffer kit (Thermo-Scientific #78833) per manufacturer's instructions using 5×10^6^–1×10^7^ cells per lysate. Protein (8–10 µg) was analyzed by SDS-PAGE, and validated using nuclear and cytoplasmic markers lamin B or GAPDH, respectively.

### Orthotopic mouse tumor model

All animal studies were approved by The Ohio State University Institutional Animal Care and Use Committee (IACUC). A375 or Hs294T human melanoma cells (2×10^6^ cells in 100 µL PBS) were injected subcutaneously into female athymic Balb/c^nu/nu^ mice as described [59]. Tumors were harvested, formalin-fixed, embedded in paraffin and sectioned for PRMT5 IHC.

### Immunoprecipitation

5×10^6^–1×10^7^ cells were lysed in MPER lysis buffer (Thermo-Scientific #78501). Lysates were pre-cleared for 30 min at 4°C on a rotating platform using True-Blot IgG beads (eBiosciences #00-8800-25; San Diego, CA). Samples were centrifuged at 10,000× *g* for 3 min, and supernatants incubated with IgG, or primary antibody against Mep50, cyclin D1, or STAT3 for 1h at 4°C rotating. Samples were incubated with IgG beads for 1h at 4°C then centrifuged at 10,000× *g* for 3 min at 4°C. Beads were washed 3× in MPER buffer, and proteins were reduced (β-mercaptoethanol) prior to immunoblot.

### Depletion of PRMT5 via siRNA

PRMT5 was depleted from cells using siRNA duplexes (Thermo Scientific, Oligo ID # ATTAA-000838;) targeting exon 15 of PRMT5 transcript: 5′-CCGCUAUUGCACCUUGGAA-3′ [15]. Silencer Select control siRNA (Ambion #4390843; Invitrogen, Carlsbad CA) was used as control. Cells were transfected using Lipofectamine 2000 (Invitrogen) per manufacturer's instructions, using OptiMEM minimal serum media (Invitrogen).

### Quantitative Real-Time PCR assay for microRNA

Forty-eight hours following transfection of melanoma cells with either control siRNA or PRMT5 siRNA (see methods), total cellular RNA was processed using TRIzol (Invitrogen) per manufacturer's instructions. Single-tube TaqMan miRNA assays for each miR of interest (Applied Biosystems) were used to detect and quantify mature miRNAs as described [60]. All data were normalized to the small nucleolar U44 RNA and expressed relative to that from cells transfected with control siRNA.

### Magnesium tetrazolium (MTT) proliferation assay

Cell proliferation was measured as optical density (O.D.) at 570 nm via MTT ((3-(4, 5-dimethylthiazolyl-2)-2, 5-diphenyltetrazolium bromide) assay; ATCC). Assays were performed in replicates of five wells/condition.

### Statistical Analysis

Fisher's exact test was used to compare the dichotomous outcome of PRMT5 protein >5% vs. ≤5% in patient specimens (localized in either the nucleus or cytoplasm) by specimen type. Wilcoxon rank sum test was used to compare percentage of PRMT5+ cells in subcellular compartments by specimen type and Spearman's correlation to evaluate relationships between staining intensity and percentage of PRMT5+ cells.

## Supporting Information

Figure S1 **PRMT5 protein does not associate with MITF protein.** IP against the MITF protein or IgG control was performed in 1106Mel and MeWo melanoma cell lines, and subjected to immunoblot using antibodies against MITF and PRMT5. The MITF antibody used here for IP recognizes both isoforms of MITF, which differ in their N-terminal regions.
(TIF)

Figure S2 **PRMT5 does not associate with BRAF or CRAF in unstimulated melanoma cells.** IP against BRAF, CRAF or IgG control was performed in representative human melanoma cell lines and subjected to immunoblot using antibodies against BRAF, CRAF or PRMT5.
(TIF)

Table S1 **Distribution of human melanoma and normal skin samples obtained for assessment of PRMT5 protein.**
(DOCX)

Table S2 **Genotype of BRAF and NRAS within human melanoma cell lines as determined by Sanger Sequencing with the ABI Prism BigDye Terminator Cycle Sequencing Kit version 3.1.**
(DOCX)
