# Supplementary figures and images for: PRMT5 Is Upregulated in Malignant and Metastatic Melanoma and Regulates Expression of MITF and p27Kip1
Source: PLoS One. 2013 Sep 30;8(9):e74710. doi: 10.1371/journal.pone.0074710 (PMC3786975; doi:10.1371/journal.pone.0074710)

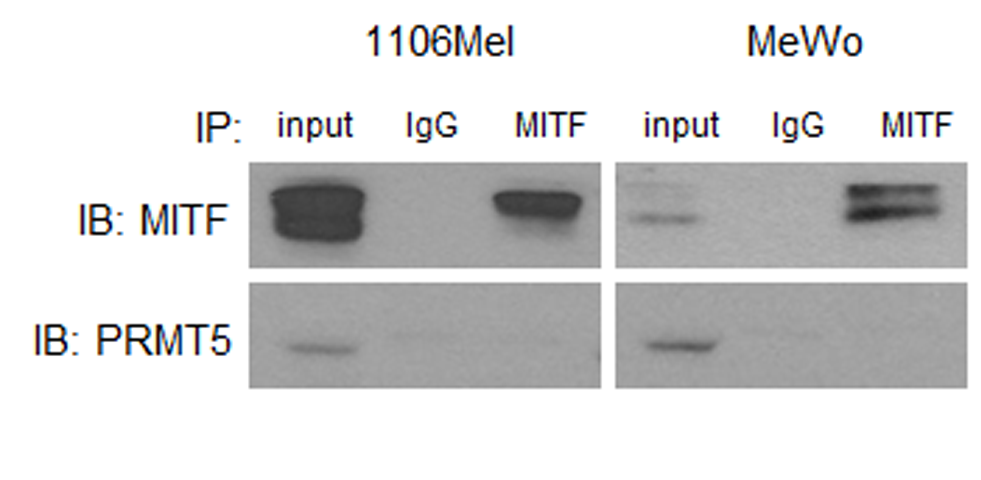

Supplement: Figure S1 — PRMT5 protein does not associate with MITF protein. IP against the MITF protein or IgG control was performed in 1106Mel and MeWo melanoma cell lines, and subjected to immunoblot using antibodies against MITF and PRMT5. The MITF antibody used here for IP recognizes both isoforms of MITF, which differ in their N-terminal regions. (TIF) [file pone.0074710.s001.tif]

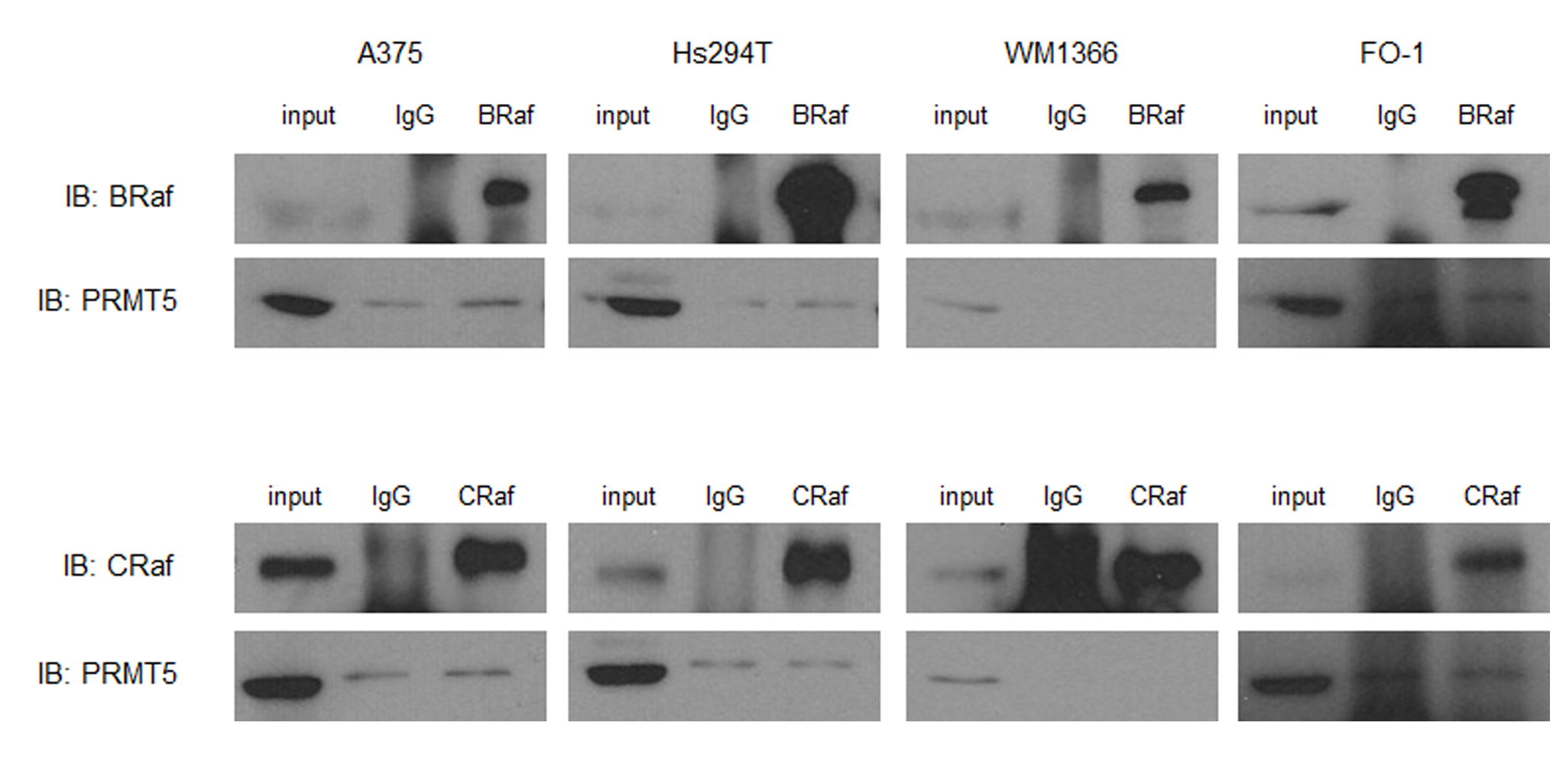

Supplement: Figure S2 — PRMT5 does not associate with BRAF or CRAF in unstimulated melanoma cells. IP against BRAF, CRAF or IgG control was performed in representative human melanoma cell lines and subjected to immunoblot using antibodies against BRAF, CRAF or PRMT5. (TIF) [file pone.0074710.s002.tif]
