# Supplementary material for: PRMT5 Is Upregulated in Malignant and Metastatic Melanoma and Regulates Expression of MITF and p27Kip1
Source: PLoS One. 2013 Sep 30;8(9):e74710. doi: 10.1371/journal.pone.0074710 (PMC3786975; doi:10.1371/journal.pone.0074710)
Supplement: Table S1 — Distribution of human melanoma and normal skin samples obtained for assessment of PRMT5 protein. (DOCX) [file pone.0074710.s003.docx]

Supplementary Table S1. Distribution of human melanoma and normal skin samples obtained for assessment of PRMT5 protein

| Total Samples | 248 |
| --- | --- |
| Male | 106 |
| Female | 99 |
| Unknown | 43 |
|  |  |
| Age Distribution (yr) | 16-94 |
| Unknown | 62 |
| Mean | 55.5 |
| Median | 55.0 |
|  |  |
|  |  |
| Normal Epidermal Tissue | 21 |
|  |  |
|  |  |
| Melanocytic Nevi |  |
| Compound nevi | 8 |
| Junctional nevi | 4 |
| Intradermal nevi | 10 |
| Unknown | 4 |
|  |  |
|  |  |
| Melanoma |  |
| *in situ* | 10 |
| Stage I | 3 |
| Stage II | 52 |
| Stage III | 8 |
| Stage IV | 23 |
| Unknown | 39 |
|  |  |
|  |  |
| Metastases |  |
|  |  |
| Lymph node | 39 |
| Brain | 1 |
| Skin | 10 |
| Lung | 5 |
| Intestine | 2 |
| Unknown | 9 |
