# Supplementary material for: PRMT5 Is Upregulated in Malignant and Metastatic Melanoma and Regulates Expression of MITF and p27Kip1
Source: PLoS One. 2013 Sep 30;8(9):e74710. doi: 10.1371/journal.pone.0074710 (PMC3786975; doi:10.1371/journal.pone.0074710)
Supplement: Table S2 — Genotype of BRAF and NRAS within human melanoma cell lines as determined by Sanger Sequencing with the ABI Prism BigDye Terminator Cycle Sequencing Kit version 3.1. (DOCX) [file pone.0074710.s004.docx]

Supplementary Table S2. Genotype of *BRAF* and *NRAS* within human melanoma cell lines as determined by Sanger Sequencing with the ABI Prism BigDye Terminator Cycle Sequencing Kit version 3.1.

| Cell Line | *BRAF* | *NRAS* |
| --- | --- | --- |
| Hs294T | V600E | WT |
| 1106Mel | V600E | WT |
| FO-1 | V600E | WT |
| WM1366 | WT | Q61L |
| CHL-1 | WT | WT |
| A375 | V600E | WT |
| MeWo | WT | WT |

Supplementary Table 3. Effects of PRMT5 siRNA on microRNA (miR) expression in human melanoma cell lines. Forty-eight hours following transfection of melanoma cells with either control siRNA or PRMT5 siRNA (see methods), total cellular RNA was processed using TRIzol (Invitrogen) per manufacturer’s instructions. Single-tube TaqMan miRNA assays for each miR of interest (Applied Biosystems) were used to detect and quantify mature miRNAs as described ([56](#_ENREF_56)). All data were normalized to the small nucleolar U44 RNA and expressed relative to that from cells transfected with control siRNA.

**Fold Change vs. Control siRNA**

| **Cell Line** | **miR-221** | **miR-222** | **miR-181b** | **miR-148a** |
| --- | --- | --- | --- | --- |
| Hs294T | 1.62 | 1.29 | 1.02 | 1.21 |
| WM1366 | 0.84 | 0.67 | 1.39 | 0.63 |
| CHL-1 | 0.42 | 0.74 | 0.68 | 2.38 |
| 1106 MEL | 1.16 | 1.42 | 2.38 | 0.72 |
